# Assessment of Ankle and Hindfoot Stability and Joint Pressures Using a Human Cadaveric Model of a Large Lateral Talar Process Excision: A Biomechanical Study: Erratum

**DOI:** 10.1097/01.md.0000464829.83065.92

**Published:** 2015-04-10

In the article “Assessment of Ankle and Hindfoot Stability and Joint Pressures Using a Human Cadaveric Model of a Large Lateral Talar Process Excision: A Biomechanical Study”,^1^ which appeared in Volume 94, Issue 11 of *Medicine*, a sentence in the Materials and Methods section has been corrected to avoid misinterpretation. The sentence in question formerly read “Center of force movement was defined as the length vector between initial and final center of force positions at 2 30-kg loading, respectively.” It now reads “Center of force movement was defined as the length vector between initial and final center of force positions at 2- and 30-kg loading, respectively.” The article has since been corrected online.
